# Hyperbolic Positioning with Antenna Arrays and Multi-Channel Pseudolite for Indoor Localization

**DOI:** 10.3390/s151025157

**Published:** 2015-09-30

**Authors:** Kenjirou Fujii, Yoshihiro Sakamoto, Wei Wang, Hiroaki Arie, Alexander Schmitz, Shigeki Sugano

**Affiliations:** 1 Hitachi Industrial Equipment Systems Co., Ltd., 3 Kanda-neribei-cho, Chiyoda-ku, Tokyo 101-0022, Japan; E-Mail: fujii-kenjirou@hitachi-ies.co.jp; 2 Department of Modern Mechanical Engineering, School of Creative Science and Engineering, Waseda University, 3-4-1 Okubo, Shinjuku-ku, Tokyo 169-8555, Japan; E-Mails: wangwei@aoni.waseda.jp (W.W.); arie@aoni.waseda.jp (H.A.); schmitz@aoni.waseda.jp (A.S.); sugano@waseda.jp (S.S.)

**Keywords:** pseudolite, hyperbolic positioning, indoor positioning, GPS

## Abstract

A hyperbolic positioning method with antenna arrays consisting of proximately-located antennas and a multi-channel pseudolite is proposed in order to overcome the problems of indoor positioning with conventional pseudolites (ground-based GPS transmitters). A two-dimensional positioning experiment using actual devices is conducted. The experimental result shows that the positioning accuracy varies centimeter- to meter-level according to the geometric relation between the pseudolite antennas and the receiver. It also shows that the bias error of the carrier-phase difference observables is more serious than their random error. Based on the size of the bias error of carrier-phase difference that is inverse-calculated from the experimental result, three-dimensional positioning performance is evaluated by computer simulation. In addition, in the three-dimensional positioning scenario, an initial value convergence analysis of the non-linear least squares is conducted. Its result shows that initial values that can converge to a right position exist at least under the proposed antenna setup. The simulated values and evaluation methods introduced in this work can be applied to various antenna setups; therefore, by using them, positioning performance can be predicted in advance of installing an actual system.

## 1. Introduction

In recent years, indoor positioning has been getting a lot of attention in both the academic and business fields. Positioning services and devices using Wi-Fi and Bluetooth low energy (BLE) have already been commercialized (e.g., Wi-Fi databases provided by Combain Mobile AB [1] and Navizon Inc. [2], and a BLE system called “iBeacon” of Apple Inc. [3]). One of the main strengths of Wi-Fi is that many access points have already been installed for communication use, and they can be directly utilized as a positioning infrastructure. On the other hand, the primary advantage of BLE, especially iBeacon, is that an API (application program interface) for iPhone, which already has a huge market share in the smartphone industry, is provided by Apple Inc. However, since Wi-Fi and BLE basically use low-accuracy positioning methods (e.g., signal strength-based trilateration (trilateration with the distances calculated by a signal propagation model) [4], fingerprinting (positioning method by matching the patterns of the acquired signal strength with a stored signal strength map) [5], and proximity detection (positioning method by detecting a reference device near the target device) [6], the positioning accuracy is limited to a few meters at best. Other indoor positioning methods that can achieve higher accuracy (such as an optical method [7], ultra-wideband (UWB) [8], and ultra-sound-based method [9]) have been proposed. However, these methods and systems are still limited to special applications because general-purpose devices such as smartphones do not include sensors necessary for them. Mautz analyzes indoor positioning technologies comprehensively and systematically in [10]. 

In Japan, a consortium consisting of the government, universities, and companies is promoting a positioning system called IMES (abbreviation of an indoor messaging system) as an indoor positioning infrastructure [11,12]. An IMES transmitter is a RFID tag that transmits a GPS-compatible signal (C/A code on the L1 band); this GPS-compatibility is one of the significant advantages of IMES because GPS is the *de facto* standard of outdoor positioning, and off-the-shelf GPS/GNSS receivers can be used with minor changes to their firmware. Since IMES uses the proximity detection as its positioning method, the system is very simple but the positioning accuracy is not high (5 to 10 m depending on the installation interval of the transmitters). 

Pseudolites are focused in this paper. Pseudolites are ground-based pseudo-satellite transmitters that were originally used to test GPS signals on the ground. In the simplest form, pseudolites transmit GPS-compatible signals similar to IMES. Since they use trilateration as its positioning method, the achievable positioning accuracy is the same level as GPS; that is, it is cm-level if the carrier phase (the phase of the carrier wave) is used and m-level if the code (modulated in the carrier wave) is used. In consideration of the GPS-compatibility and the positioning accuracy, pseudolites can be expected to be a next generation of IMES. In other words, they have a potential to be a high-accuracy indoor positioning infrastructure.

**Figure 1:**
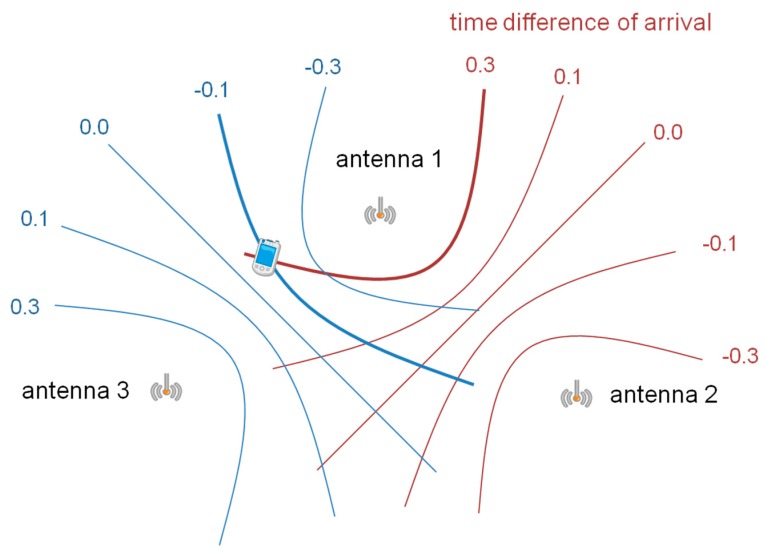
Concept of hyperbolic positioning.

The present work is an extension of our previous work [13]. In the previous work, three problems of pseudolites (near-far, synchronization, and integer ambiguity resolution) were introduced, and in order to overcome these problems, a hyperbolic positioning method with closely-located pseudolite antennas was proposed. Hyperbolic positioning, which is also called “multilateration,” is a common technique used for positioning when the absolute time reference does not exist. Figure 1 explains the positioning method of a two-dimensional positioning scenario; if the time difference of arrival of signals from two transmitters is constant, the receiver’s position is on a hyperbola; if there are three or more transmitters, the intersection of hyperbolas obtained from two or more pairs of transmitters is the receiver’s position. 

In the previous work, a two-dimensional positioning experiment was conducted based on the proposed hyperbolic positioning method, and its positioning accuracy was evaluated. In the present work, in addition to introducing the previously-proposed positioning theory and the experimental result of two-dimensional positioning, the cause of the positioning error is analyzed in more detail, and the performance of three-dimensional positioning is evaluated with a computer simulation. For the proposed method and system, three-dimensional positioning is not a simple extension of the two-dimensional one unlike other positioning systems such as GPS trilateration and Wi-Fi fingerprinting because the positioning accuracy and the initial value convergence of the non-linear least squares used for position calculation are very sensitive to the geometry of the antenna setup of the transmitter. Accordingly, in the present work, two types of antenna arrays with different geometric setups are evaluated, and how the initial value converges to the proper position is analyzed. Since these evaluation methods can be applied to various pseudolite setups, they can be used to know the positioning performance of a designed system in advance of installing it. Moreover, they can also be used to make a guideline of the antenna setup; this is important for a positioning infrastructure.

## 2. Positioning Theory

### 2.1. Overview

**Figure 2:**
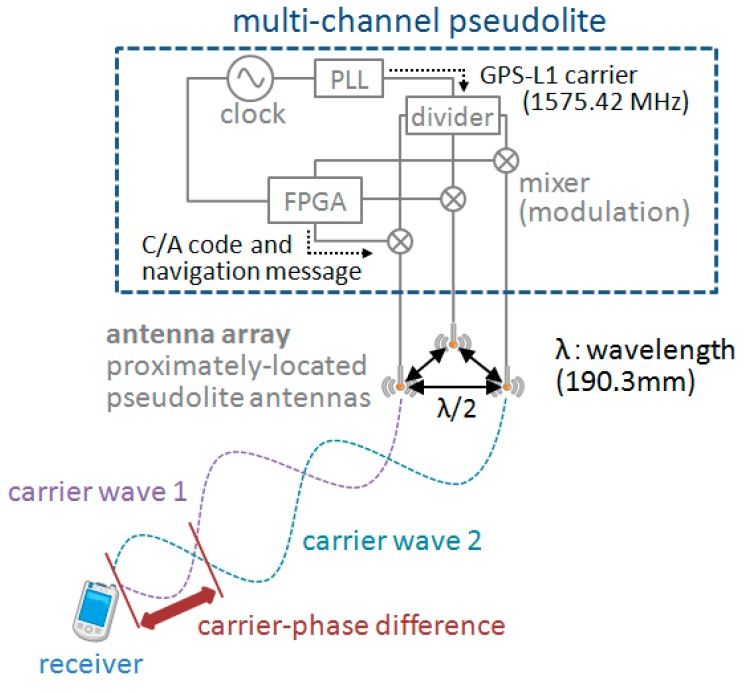
Overview of multi-channel pseudolite and carrier-phase difference acquisition.

A schematic of the multi-channel pseudolite is given in Figure 2. As shown in the figure, the antenna array consists of multiple/3 pseudolite antennas. The antennas are located at intervals of a half-wavelength of a common GPS L1 carrier wave, *i.e.*, at 95.15 mm to each other. Each antenna transmits a signal with a different C/A code (which is used to distinguish channels) and a navigation message (which includes the ID or position of the antenna) by modulating them on a GPS L1 carrier wave. Since the carrier waves transmitted from the antennas are all generated by a single phase-locked loop (PLL), their wavelength and frequency are the same; accordingly, the difference between the phases of each pair of received carrier waves (*i.e.*, carrier-phase difference, or CPD, hereafter) does not vary as long as the receiver remains still at the same position, and it changes when the receiver moves to a different position. Since the CPD of each pair of the antennas is constant on the same hyperbolic line similar to the concept shown in Figure 1, the intersection of hyperbolic lines is the receiver’s position.

### 2.2. Positioning Algorithm

Normally, a GPS/GNSS receiver outputs a carrier phase as an observable, which is an integrated value of the number of whole cycles and the fraction of a cycle of a beat wave that arises between the received and receiver-generated carrier waves. If the carrier phase corresponding to pseudolite antenna *k* is represented as *φ^k^* (whose unit is cycles), it is modeled as

(1) $$\phi^{k}=\lambda^{-1}\left\|{r^{k}}_{t}-r_{u}\right\|+\lambda^{-1}c(\delta t-\delta T)-N^{k}+{\varepsilon^{k}}_{\phi }$$

where *λ* is the wavelength of GPS L1-band, **r***_t_^k^* is the position of the pseudolite antenna *k* (which is known), **r***_u_* is the receiver position to be determined, *c* is the speed of light, *δt* is the clock bias of the receiver, *δT* is the clock bias of the multi-channel pseudolite system, *N^k^* is the integer ambiguity (which is an integer value that consists of the number of wave fronts existing between the transmitter and receiver antennas and an integrated value of cycles of the beat wave mentioned above; see also [14]), and *ε^k^_φ_* is the observation error of the carrier phase. This kind of equation modeling the observable by using the geometric relation between the receiver and transmitter is called “observation equation” in the terminology of GPS/GNSS. Since the signals transmitted from the antennas are synchronized, the subtraction of Equation (1) for antenna *k* from that for antenna *l* gives

(2) $$\phi^{lk}=\phi^{l}-\phi^{k}=\lambda^{-1}(\left\|{r^{l}}_{t}-r_{u}\right\|-\left\|{r^{k}}_{t}-r_{u}\right\|)-N^{lk}+{\varepsilon^{lk}}_{\phi }$$

where *φ^lk^* is the carrier-phase difference (CPD), and *N^lk^* (*i.e.*, *N^l^* − *N^k^*) is constant over time if the receiver remains still. Note that since the carrier waves transmitted from the antennas *l* and *k* have the same frequency by sharing a common PLL as shown in Figure 2, the integrated values of the beat wave cycles included in *N^l^* and *N^k^* are the same, and they are cancelled out by the subtraction in Equation (2); accordingly, *N^lk^* varies depending on only the geometry of transmitter and receiver antennas.

Here, if only the fractional part of *φ^lk^* is used as the observable, and the absolute value of $\lambda^{-1}\left(\left\|r_{t}^{l}-r_{u}\right\|-\left\|r_{t}^{k}-r_{u}\right\|\right)$ is limited to less than 0.5 (*i.e.*, the distance between pseudolite antennas *l* and *k* is less than a half wavelength), *N^lk^* is always zero; as a result, the integer ambiguity does not need to be resolved. Equation (2) can therefore be reduced to

(3) $$\lambda \phi^{lk}=\left\|{r^{l}}_{t}-r_{u}\right\|-\left\|{r^{k}}_{t}-r_{u}\right\|+{\varepsilon^{lk}}_{\lambda \phi }$$

This is the observation equation of the proposed hyperbolic positioning method. Both the left-hand side (observable) and right-hand side (model) are caused by the difference between the travel distances of the carrier waves from the antennas *l* and *k* (for convenience, this is also called carrier-phase difference or CPD hereafter if not otherwise specified).

When the CPD (*φ^lk^*) is calculated from two carrier-phase outputs from the receiver, phase inversion of each carrier wave has to be considered because GPS receivers can track a received carrier wave even if it is inverted 180 degrees because of the modulation of a navigation message. Phase inversion can be detected by checking the 29th or 30th bit of the handover word (HOW) of the navigation message, which must always be zero according to the GPS specification [15]. If the received carrier wave is inverted, 0.5 must be added to its phase to revert it.

If the receiver position to be estimated is three dimensional, three or more linearly independent observation equations (Equation (3)) are necessary; in that case, four or more pseudolite antennas are needed, and at least one of them has to be placed in a different plane from the others. If the position to be estimated is two dimensional, three or more antennas are necessary, and at least one of them must be located on a different line from the others.

Since the observation equation is non-linear, a non-linear least-square method (the Newton-Raphson method) is used to determine the receiver position. If the non-linear term in Equation (3) is defined as

(4) $$F^{lk}(r_{u})=\left\|{r^{l}}_{t}-r_{u}\right\|-\left\|{r^{k}}_{t}-r_{u}\right\|$$

its partial derivative with respect to **r***_u_* is

(5) $$\frac{\partial F^{lk}(r_{u})}{\partial r_{u}}=-\frac{{\left({r^{l}}_{t}-r_{u}\right)}^{\text{T}}}{\left\|{r^{l}}_{t}-r_{u}\right\|}+\frac{{\left({r^{k}}_{t}-r_{u}\right)}^{\text{T}}}{\left\|{r^{k}}_{t}-r_{u}\right\|}$$

If the initial value of **r***_u_* used for the solution-updating process of the Newton-Raphson method is described as **r***_u,_*_0_ = (x_0_, y_0_, z_0_), and if the second- and higher-order terms of the Taylor expansion of *F^lk^*(**r***_u,_*_0_) are ignored, the first updated solution is represented as

(6) $$F^{lk}(r_{u,1})\approx \frac{\partial F^{lk}(r_{u,0})}{\partial r_{u,0}}\Delta r_{u,0}+F^{lk}(r_{u,0})$$

Equation (3) is therefore modified to

(7) $$\begin{array}{ll} \lambda \varphi^{lk} & =F^{lk}(r_{u,1})+\varepsilon_{\lambda \varphi }^{lk}\\ & \approx \frac{\partial F^{lk}(r_{u,0})}{\partial r_{u,0}}\Delta r_{u,0}+F^{lk}(r_{u,0})+\varepsilon_{\lambda \varphi }^{lk}. \end{array}$$

Here, if the number of antennas connected from a multi-channel pseudolite is *m* (the proposed method assumes that *m* is 3 and more, although only three antennas are depicted in Figure 2), the number of linearized observation equations (Equation (7)), *n*, is

(8) $$n={}_{m}C_{2}=\frac{m!}{2!(m-2)!}$$

where *_m_*C_2_ is the number of 2 combinations from *m* elements. Those observation equations are expressed in the following matrix form as

(9) $$\left[\begin{array}{ccc} \frac{\partial F_{0}^{21}}{\partial x_{0}} & \frac{\partial F_{0}^{21}}{\partial y_{0}} & \frac{\partial F_{0}^{21}}{\partial z_{0}}\\ & \vdots & \\ \frac{\partial F_{0}^{1n}}{\partial x_{0}} & \frac{\partial F_{0}^{1n}}{\partial y_{0}} & \frac{\partial F_{0}^{1n}}{\partial z_{0}} \end{array}\right]\left[\begin{array}{c} \Delta x_{0}\\ \Delta y_{0}\\ \Delta z_{0} \end{array}\right]=\left[\begin{array}{c} \lambda \varphi^{21}-F_{0}^{21}\\ \vdots \\ \lambda \varphi^{1n}-F_{0}^{1n} \end{array}\right]+\left[\begin{array}{c} \varepsilon_{\lambda \varphi }^{21}\\ \vdots \\ \varepsilon_{\lambda \varphi }^{1n} \end{array}\right].$$

The matrix on the left-hand side of Equation (9) is defined as **G**, called the “geometry matrix”, and the two column vectors on the right-hand side are respectively defined as **b** (left one) and **ε***_λφ_* (right one; *i.e.*, observation noise). Equation (9) is then expressed as

(10) $$G\Delta r_{u,0}=b+\varepsilon_{\lambda \varphi }$$

If the estimated value of Δ**r**_*u,*0_ is denoted as $\Delta {\hat{r}}_{u,0}$, the solution to Equation (10) is given as

(11) $$\Delta {\hat{r}}_{u,0}={\left(G^{\text{T}}G\right)}^{-1}G^{\text{T}}b$$

The estimated position is then updated iteratively according to

(12) $${\hat{r}}_{u,1}=r_{u,0}+\Delta {\hat{r}}_{u,0}$$

After this updating process is repeated several times, a sufficiently approximate solution for the receiver position, ${\hat{r}}_{u}$, is obtained.

### 2.3. Dilution of Precision

As in the case of outdoor GPS/GNSS, the magnitude of the positioning error is estimated by the so-called “dilution of precision” (DOP) [16]. In order to calculate the DOP of hyperbolic positioning, a standard deviation of CPDs of carrier waves (shown as the left-hand side of Equation (3)) is used. If it is denoted as *σ_λφ_*, the covariance matrix of Δ**r***_u_* is given as

(13) $$\text{cov}(\Delta r_{u})=\sigma_{\lambda \phi }^{2}{\left(G^{\text{T}}G\right)}^{-1}$$

If (**G**^T^**G**)^−1^ is defined as **H**, the DOP is expressed as the diagonal elements of **H**, where

(14) $$\text{H}=\left\lceil \begin{array}{ccc} \text{XDO}{\text{P}}^{2} & • & •\\ • & \text{YDO}{\text{P}}^{2} & •\\ • & • & \text{ZDO}{\text{P}}^{2} \end{array}\right\rceil$$

Here, “XDOP” means the DOP for the *x*-coordinate (likewise, for the *y*- and *z*-coordinates). From Equations (13) and (14), the variance of positioning error for each *x*-, *y*-, and *z*-coordinate is given by

(15) $$\sigma_{x}^{2}=\sigma_{\lambda \phi }^{2}\text{XDO}{\text{P}}^{2}$$

(16) $$\sigma_{y}^{2}=\sigma_{\lambda \phi }^{2}\text{YDO}{\text{P}}^{2}$$

(17) $$\sigma_{z}^{2}=\sigma_{\lambda \phi }^{2}\text{ZDO}{\text{P}}^{2}$$

If the DOP for the *x*-*y* plane is defined as HDOP,

(18) $$\text{HDO}{\text{P}}^{2}=\text{XDO}{\text{P}}^{2}+\text{YDO}{\text{P}}^{2}$$

Moreover, if the standard deviation of the estimated position on the *x*-*y* plane, *σ_xy_*, is defined as

(19) $$\sigma_{xy}=\sqrt{\sigma_{x}^{2}+\sigma_{y}^{2}}$$

from Equations (15)−(19), *σ_xy_* can be expressed as

(20) $$\sigma_{xy}=\sigma_{\lambda \phi }\text{HDOP}$$

Likewise, if the DOP for the three-dimensional space is defined as PDOP,

(21) $${\text{PDOP}}^{2}={\text{XDOP}}^{2}+{\text{YDOP}}^{2}+{\text{ZDOP}}^{2}$$

Then, the standard deviation of the estimated three-dimensional position, *σ_xyz_*, is defined as

(22) $$\sigma_{xyz}=\sqrt{\sigma_{x}^{2}+\sigma_{y}^{2}+\sigma_{z}^{2}}=\sigma_{\lambda \phi }\text{PDOP}$$

Equations (20) and (22) mean that *σ_xy_* and *σ_xyz_* can be deduced from the standard deviation of the CPDs and the geometric relation between the transmitters and receiver. In other words, Equations (20) and (22) are the functions to convert the error of the observable to a positioning error.

## 3. Two-Dimensional Positioning Experiment

### 3.1. Devices

**Figure 3:**
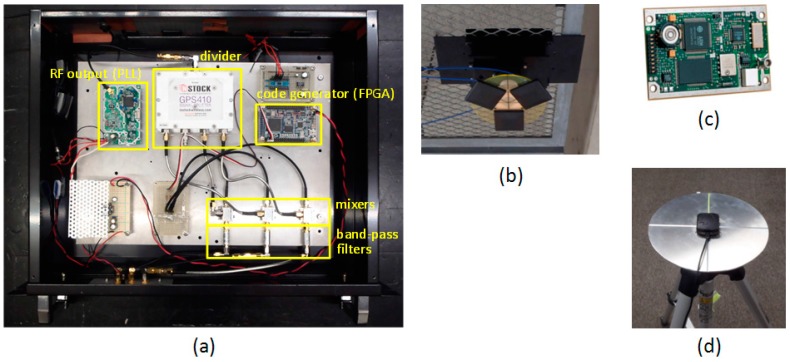
(**a**) Multi-channel pseudolite (three channels); (**b**) Three channel antenna array; (**c**) SuperStar II^TM^ receiver module; (**d**) Receiver antenna on ground plane.

A prototype of multi-channel (three-channel) pseudolite is shown in Figure 3a. Its architecture is largely consistent with the diagram depicted in Figure 2; a GPS L1 carrier wave (1575.42 MHz) generated by a PLL is divided into three, and each wave is modulated by a digital signal (composed of a C/A code with a transmission rate of 1.023 Mbps and a navigation message with a transmission rate of 50 bps) created by a field-programmable gate array (FPGA). Since only one PLL is used, the CPDs are always constant, both during the operation of the pseudolite and when it is switched on after being switched off. The multi-channel pseudolite connects to an antenna array consisting of three antennas shown in Figure 3b. Patch antennas (PA175-S of Allis Communications Co., Taipei, Taiwan) are located at the interval of the half-wavelength of the GPS L1 carrier wave, *i.e.*, at 95.15 mm from each other. As the receiver, a SuperStar II^TM^ from NovAtel Inc. (Calgary, Canada), with firmware modified so that pseudolite signals can be received, is used (Figure 3c). The receiver antenna is mounted on a ground plane with a 200 mm diameter (Figure 3d) to avoid the multipath propagation from the floor.

### 3.2. Setup and Procedure

**Figure 4:**
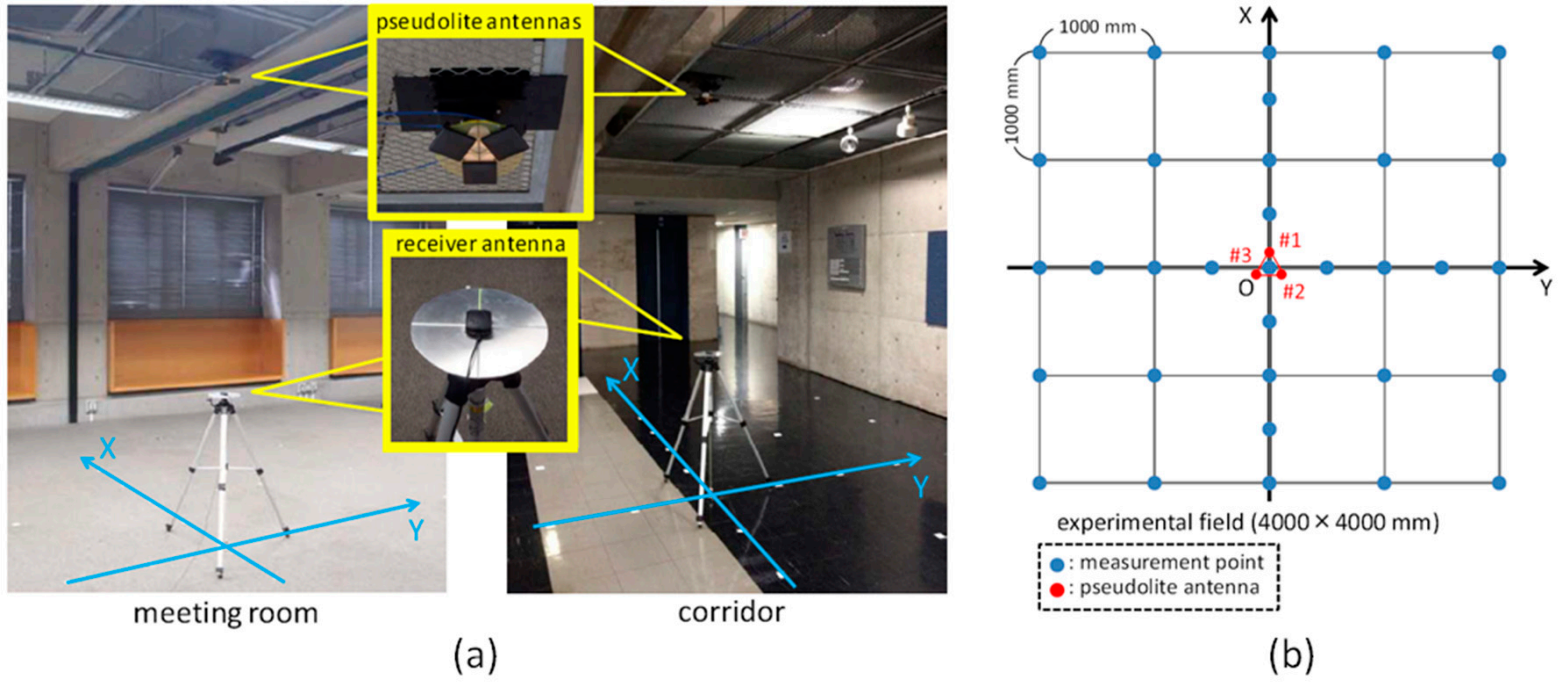
(**a**) Appearance of experimental fields; (**b**) Measurement points of CPD and positions of pseudolite antennas on horizontal plane.

In order to evaluate the two-dimensional hyperbolic positioning with the prototype multi-channel pseudolite, two positioning experiments were conducted in different places, a meeting room and a corridor (Figure 4a). The same experimental setup was applied to both places (except the height of the pseudolite antenna array); Figure 4b shows the positions in which the CPD was measured and in which the pseudolite antennas were installed in the given coordinate system (four-meter square area). In each place, as seen in Figure 4b, the CPD was measured at a total of 33 points; on the *x*- and *y*-axes, it was measured at 500-mm intervals; otherwise, it was measured at 1000-mm intervals on the grid. As for the antenna array, three pseudolite antennas (#1, #2, and #3) were placed at the vertices of a regular triangle with a side length of 95.15 mm (a half wavelength of the carrier wave), drawn on a circular plate. This antenna array was attached to the ceiling in such a way that the center of gravity of the triangle was exactly above the origin of the coordinate system, and antenna #1 was on the *x*-axis. (For convenience, the origin of the coordinate system is simply called the “origin”, hereafter.) The inclination of the antenna array was carefully adjusted to zero (horizontal). The height of the antenna array was set to 2832 mm in the meeting room and 2546 mm in the corridor, and the height of receiver antenna was set to 944 mm in both places.

In consideration of the setup above, CPDs for all combinations of pseudolite antennas must be zero at the origin because the distances between the receiver and pseudolite antennas are the same. For this reason, the CPDs were calibrated by using phase-shifters inserted between the output connectors of the multi-channel pseudolite and the input ones of the antenna array so that all the CPDs become zero at the origin.

The carrier-phase was measured for 60 s (120 epochs with the receiver’s sampling rate of 2 Hz) at each measurement position. After the measurements at all 33 measurement positions were completed, the two-dimensional positions of the receiver were estimated by using the algorithm described in Section 2.2.

### 3.3. Experimental Results

**Figure 5:**
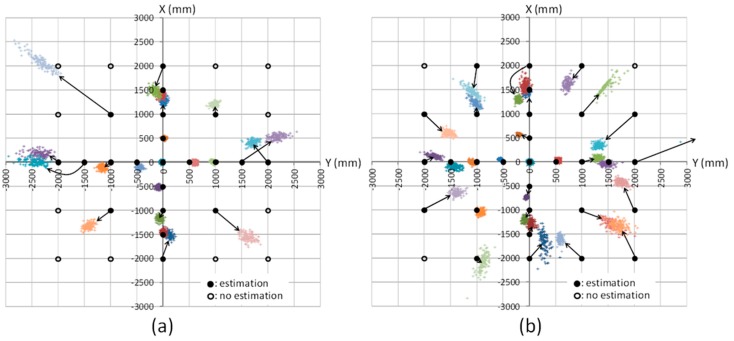
Results of position estimation at each measurement point: (**a**) meeting room and (**b**) corridor.

The results of the position estimation conducted for each epoch at each measurement point are plotted in Figure 5a (meeting room) and 5b (corridor). The solid black circles represent the measurement positions where position estimation could be done; the empty black circles represent the positions where position estimation could not be done because the receiver could not obtain all three observables (*i.e.*, CPDs shown in the left-hand side of Equation (3)) necessary for the position estimation. The black arrows represent the correspondence between the estimated positions and the true position. As seen in those figures, the positioning accuracy is relatively good around the origin but, as the distance between measurement position and the origin increases, the positioning accuracy decreases. The positioning errors that occur depending on the measurement positions are not biased to the same direction; rather, as seen from the black arrows in the figures, the direction of error occurrence appears to be almost random.

**Figure 6:**
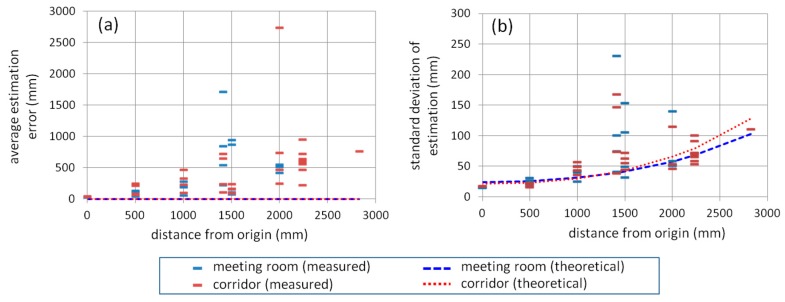
Relation between positioning error on x-y plane at each measurement position and distance of measurement position from the origin of coordinate system: (**a**) average of position estimation error and (**b**) standard deviation of estimated position.

The position estimation results shown in Figure 5 are summarized in Figure 6 as follows. The position estimation error on the *x*-*y* plane, *E_xy_*, is calculated for each epoch by using $E_{xy}=\sqrt{E_{x}^{2}+E_{y}^{2}}$ where *E_x_* and *E_y_* are the positioning errors on *x*- and *y*-coordinate, respectively. Then, its average and standard deviation at each measurement position in the meeting room and corridor are calculated and plotted in Figure 6a (average) and 6b (standard deviation) in relation to the distance between the origin and measurement position on the *x*-*y* plane. In both figures, the theoretical values of average estimation error and standard deviation of estimation are also represented; the theoretical average estimation error is set to zero assuming no setup bias and no bias error in the CPD observables, and the theoretical standard deviation is calculated from Equation (20) based on the geometric relation between the pseudolite antennas and the receiver with the value of *σ_λφ_* of 1.04 mm which is obtained by averaging the one-minute measured values at the origin.

As seen in the both graphs in Figure 6, when the measurement position is distant from the origin, both the average estimation error and standard deviation increase, although some outliers (data with large error) are seen at the distance of around 1400 and 2000 mm. Note that there was a huge outlier at the distance of 2000 mm in Figure 6b (its standard deviation is 1478 mm) that is not shown in the figure because its scale is too large compared to that of the others. If those outlier-like values are ignored, as can be seen in Figure 6b, the theoretical standard deviation is largely consistent of the actual measured values; on the other hand, as seen in Figure 6a, relatively large gaps exist between the theoretical average error and actual values. These graphs also show that the magnitude of the average estimation error is about 10 times more than that of the standard deviation; that is, the average estimation error has larger impact for positioning performance. As a result, from Figure 6a, the achieved positioning accuracy with the developed system based on the proposed positioning method is centimeter- to meter-level.

### 3.4. Discussion

As shown in Equation (20), the standard deviation of position estimation (*i.e.*, random error of the estimated position) derives from the random error of CPD. This is the reason that the actual values were roughly consistent to the theoretical values in Figure 6b. 

On the other hand, there are two possible reasons why the average position estimation error (*i.e.*, bias error of the estimated position) is large compared to the theoretical values as shown in Figure 6a. One of them is a multipath propagation of the carrier wave. In the experimental fields, there were a lot of materials that reflect radio waves, such as wire mesh on the ceiling and large metallic partitions. As the distance of the receiver from the pseudolite antennas increases, the risk of multipath propagation also increases, because the elevation angle of the pseudolite antennas with respect to the receiver antenna decreases, and the propagation distance of the radio wave increases; as a result, the receiver easily receives reflected waves. This could be the reason that the positioning bias error was relatively large at the distance of around 1400 and 2000 mm in Figure 6a, especially the reason of the huge error such as the ones of about 1700 and 2750 mm.

The multipath propagation also causes the interference between the direct and reflected waves (so-called multipath interference). When this occurs, the receiver cannot acquire the direct wave correctly. This is likely the reason that there were some measurement positions, especially distant from the origin, in which the carrier phase could not be acquired in the meeting room (shown as empty circles in Figure 5a) but it could be acquired in the same measurement positions of the corridor (shown as filled circles in Figure 5b).

The other possible cause of the bias error is the so-called “antenna phase-centre variation” (PCV). The radio-wave emission point or receiving point (phase center) of an antenna is usually different from the physical center of the antenna. Moreover, the position of the phase center frequently varies in millimeters to centimeters according to the geometric relation between the receiver and transmitter antennas [17]; this would be the reason that the direction of the positioning bias error (shown as black arrows in Figure 5) is almost random. The scale of the carrier-phase error caused by the PCV (mm to cm) is far larger than the random error of the CPD (1.04 mm).

The occurrence of the positioning bias error (caused by the multipath propagation and the PCV) means that the geometric relation between the pseudolite antennas and the receiver antenna are virtually distorted. This influences also the random error of estimated position because the error magnitude depends on the geometry; this is the reason that, in Figure 6a,b, the average error and standard deviation at the distance of around 1400 and 2000 mm are both large.

## 4. Preparation for Simulation

As implicitly assuming in the discussion above, the error of the CPD expressed as *σ_λφ_* in Equation (20) includes two components: bias and random errors. From the experimental results mentioned in Section 3.3, the bias error of estimated position is about 10 times larger than its random error; this means that the bias error of the CPD is also 10 times larger than its random error. In this section, the bias error of the CPD is inverse-calculated from the experimental results shown above so that the calculated value can be used for the simulation introduced in the next section.

**Figure 7:**
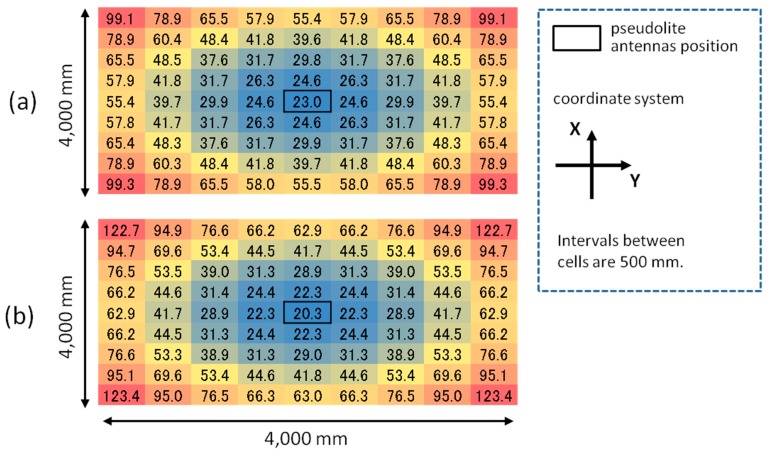
HDOP distributions (top views): (**a**) meeting room and (**b**) corridor.

First of all, the HDOP described in Equation (20) is calculated for each point of the 500-mm square grid of the four-meter square area for each experimental setup (both the meeting room and the corridor). A HDOP value in a grid point can be calculated by using Equations (3)–(18) combined with a value of the ideal CPD calculated by the geometric relation between the pseudolite antennas and the receiver. The HDOP values for each setup of the meeting room and corridor are respectively shown in Figure 7a,b. As seen in the figures, the value of HDOP is the minimum at the position just below the pseudolite antenna array.

**Figure 8:**
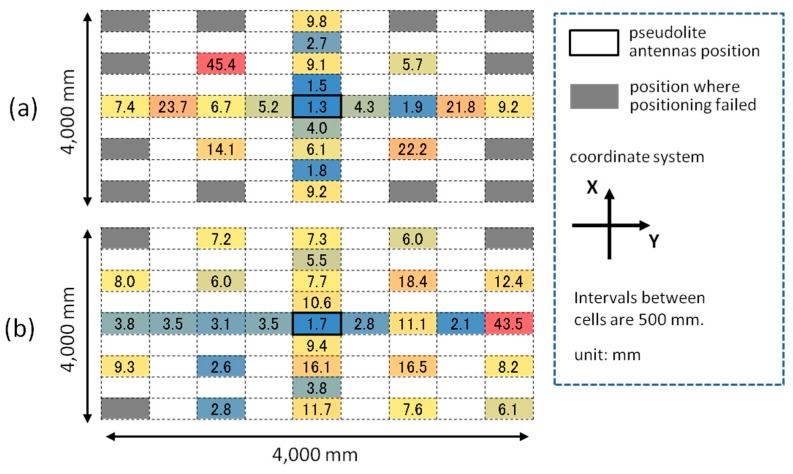
Estimated observation error of CPD: (**a**) meeting room and (**b**) corridor. (Unit is in millimeter)

**Figure 9:**
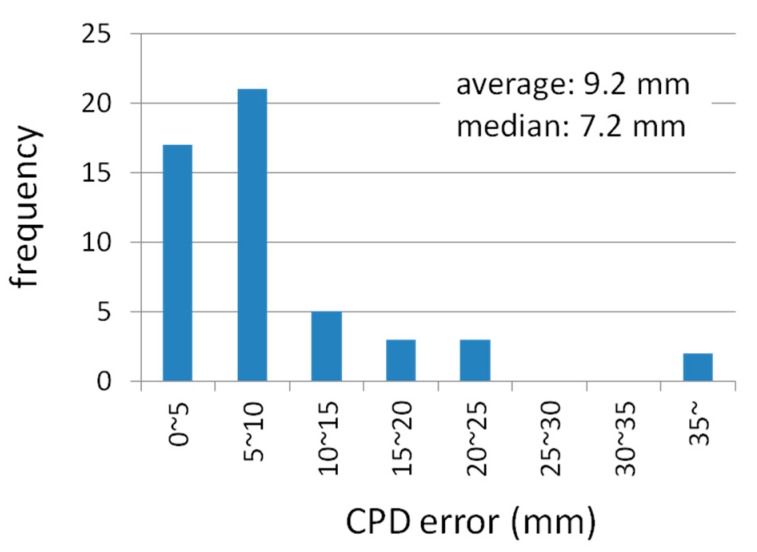
Histogram of estimated observation error of CPD for both meeting room and corridor.

According to Equation (20), if the positioning error on the *x*-*y* plane shown in Figure 5 and Figure 6a are divided by the HDOP values in Figure 7, the error of the CPD, *σ_λφ_*, is inversely calculated (in millimeter). Figure 8 and Figure 9 respectively show the calculation result and its histogram. The average and median values of the CPD error are 9.2 and 7.2 mm, respectively. These values are not so different from the results of PCV analysis shown in [17], which mentions that the value of PCV varies within a few centimeters. Accordingly, these values can be used as the basic data of the computer simulation to evaluate the positioning performance of various pseudolite setups.

## 5. Simulation of Three-Dimensional Positioning

In this section, a computer simulation of three-dimensional positioning is conducted based on the positioning theory introduced in Section 2; especially, the positioning performance under the occurrence of antenna PCV is evaluated by using the value of CPD error derived in Section 4. The primary difference between two- and three-dimensional positioning is the antenna arrangement; to achieve the three-dimensional positioning, four or more pseudolite antennas must be placed on different planes so that the rank of matrix **G** in Equation (10) becomes three.

**Figure 10:**
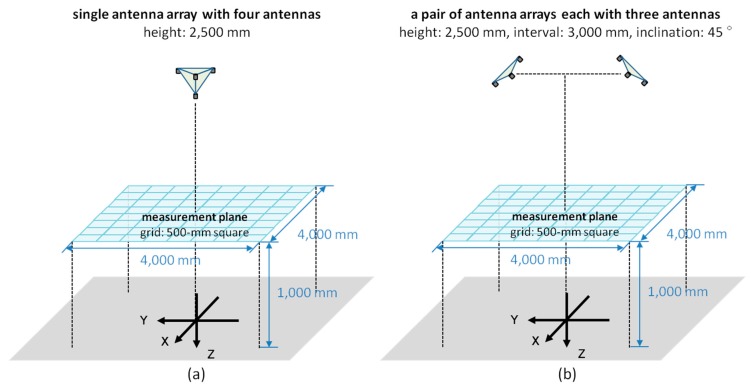
Simulation setup with (**a**) single antenna array and (**b**) double antenna arrays.

Two types of antenna arrangement are supposed here: one is the simplest form with a single array of four pseudolite antennas shown in Figure 10a and the other is the second possibly simplest one with a pair of antenna arrays each with three pseudolite antennas shown in Figure 10b (for convenience, the former is called “single array” and the latter is called “double array”, hereafter). The four antennas of the single array are attached to the vertices of a regular tetrahedron with the edge of a half wavelength of the GPS L1 carrier (95.15 mm). Each of the antenna arrays of the double array is the same as that used for two-dimensional positioning described in the previous sections. The antenna setup consisting of three antenna arrays each with two antennas (triple array) is also simple but the double array is better for practical use because it can be also used alone if only two-dimensional positioning is required.

Figure 10a,b also shows the simulation setup. Similar to the experimental setup mentioned in Section 3, the size of the measurement area is 4000-mm square and the measurement positions are 81 points on the grid of 500 mm. The height of the single array (top plane consisting of three antennas) and that of the double array (the height of the center of gravity of each antenna array) are both set at 2500 mm. In the case of double array, two antenna arrays are 45-degree inclined so that the vertical line from the center of the gravity of each antenna array faces to the center of measurement area. In addition, two antenna arrays are separated with a 3000-mm distance; this distance does not have a particular meaning but it is assumed that the antenna arrays are installed at a building entrance or an intersection of corridors. Moreover, since the distance of 3000 mm is not long compared to the antenna height, the near-far problem is not an issue. Furthermore, also the cabling is not an issue, because the two antenna arrays do not need to synchronize to each other; that is, two independent pseudolite sets can be used. The initial value of the non-linear least squares, $r_{u,0}$, shown in Equations (6) and (12) is set to the true position of the receiver so that the position calculation is successfully done; as analyzed in the next section, the position calculation does not necessarily converge to a right solution depending on the initial value.

**Figure 11:**
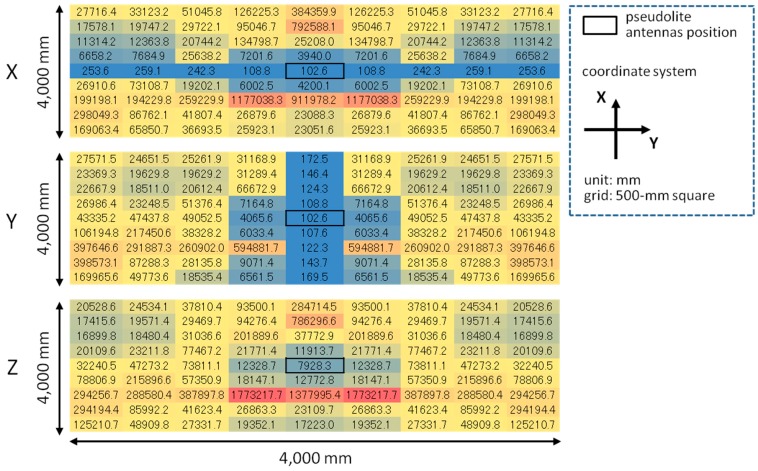
Error simulation results for each of *x*-, *y*-, and *z*-coordinates with single array.

**Figure 12:**
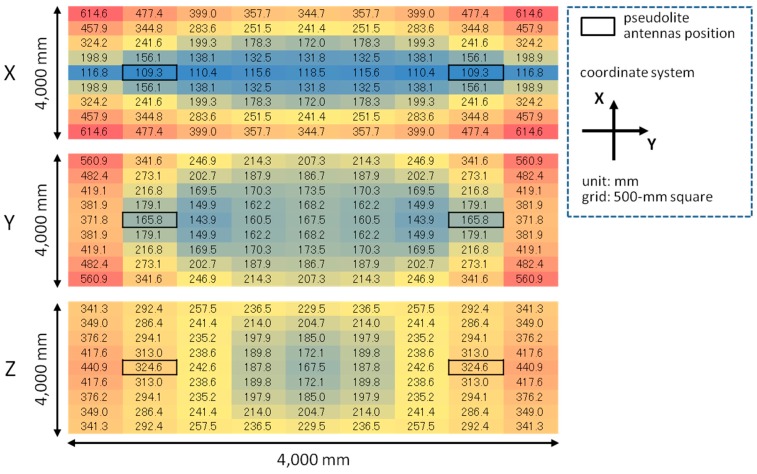
Error simulation results for each of *x*-, *y*-, and *z*-coordinates with double array.

**Figure 13:**
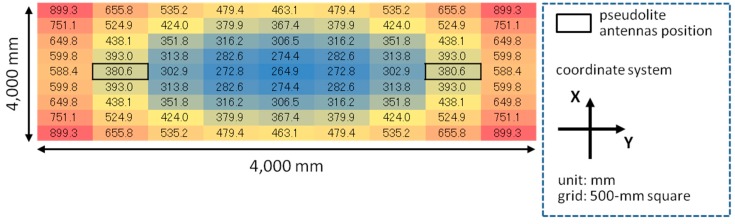
Three-dimensional positioning error with double array based on CPD bias error (9.2 mm).

**Figure 14:**
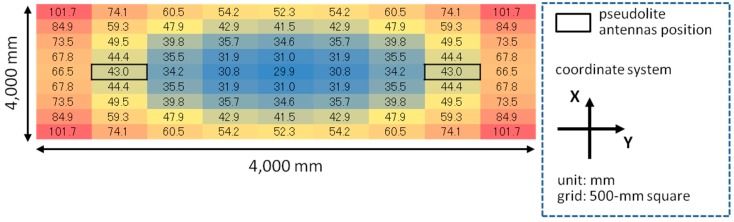
Three-dimensional positioning error with double array based on CPD random error (1.04 mm).

The error of three-dimensional positioning for each *x*, *y*, and *z*-coordinate was simulated using Equations (15)–(17) based on the geometric relation between pseudolite antenna arrays and measurement positions (receiver antenna positions) shown in Figure 10 and the average CPD error of 9.2 mm derived in Section 4. Figure 11 and Figure 12 respectively show the positioning error for the case of single array and double array. As can be seen in Figure 11, in the case of single array, the positioning error is huge other than on the *x*- and *y*-axes. On the other hand, in the case of double array shown in Figure 12, the positioning error is small over the measurement area, especially in the area surrounded by the two antenna arrays. The three-dimensional positioning error calculated from $\sigma_{xyz}=\sqrt{\sigma_{x}^{2}+\sigma_{y}^{2}+\sigma_{z}^{2}}$ in the case of double array is shown in Figure 13. As seen in the figure, the positioning error around the center of the measurement area is decimeter-level. If the bias error stemmed from the antenna PCV is ignored (assuming the use of ideal antennas) and only the random error of the CPD of 1.04 mm influences the position estimation, the positioning errors become those shown in Figure 14. Similar to the experimental results shown in Figure 5, the scale of the error of the bias error is about ten times more than that of the random error.

## 6. Initial Value Convergence Analysis

In the two-dimensional positioning experiment mentioned in Section 3, the initial value for the non-linear least squares (**r***_u,_*_0_ of Equations (6) and (12)) was set to the position of the pseudolite antenna array (*i.e.*, the center of gravity of three antennas), and it always converged to the right position as long as the carrier-phases were obtained rightly, although a certain amount of positioning error occurs. However, in the simulation using the double array described in Section 5, initial values around pseudolite antennas often did not converge to a right position (this is the reason that, in the simulation, the initial value is always set to the true position of the receiver). In this section how the initial value converges to a right position in the case of using the double array is analyzed. 

The simulation setup is the same as in the case of the double array in the previous section (4000-mm square field and 81 measurement points on the 500-mm grid). Whether the initial value can converge to a right position or not is investigated for each of the 81 measurement points while varying the *z*-coordinate of the initial value from −2500 (the height of the double array) to 0 (ground) with the step of 250 mm keeping the *x*- and *y*-coordinates at 0 (the midpoint between two antenna arrays). Basically, the initial value converges to a position close to the correct position or diverges to a huge value (clearly not a pseudo-solution such as a local minimum); therefore, it is simple to judge if the converged value is right or not.

**Figure 15:**
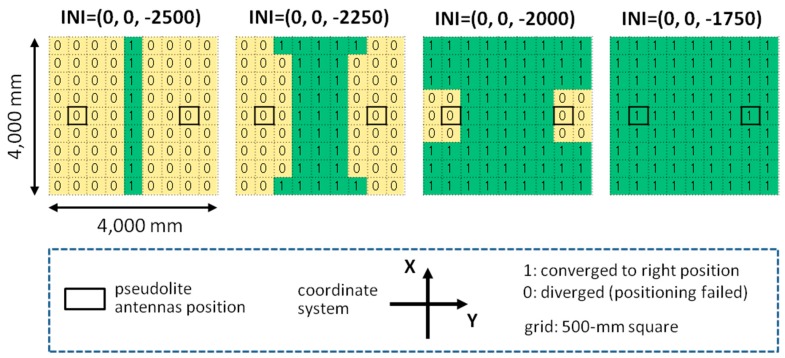
Results of initial value convergence.

Figure 15 shows the results of the initial value convergence. The results with the *z*-coordinate of less than −1750 are omitted because they are the same as that of the case of −1750; that is, the initial value converges to the right positions in all measurement points when the height is less than 1750 mm. These results give a guideline of how to set an initial value (e.g., in the case of using double array, it should be set to the midpoint between two antennas on the ground). However, since the right initial value could change according to the antenna setup, an analysis similar to this work is necessary for a different setup.

## 7. Conclusions

This work introduces a novel use of pseudolites to cope with pseudolites’ conventional problems on the indoor positioning. The experimental results of two-dimensional positioning with a single antenna array show that the positioning accuracy varies from centimeter- to meter-level according to the geometric relation between the antenna array and the receiver. In addition, these results suggest that the antenna PCV is critical for the positioning accuracy. Based on the error of CPD inverse-calculated from those experimental results, a simulation of three-dimensional positioning is conducted. The simulation result suggests that positioning with two antenna arrays can achieve a relatively good accuracy. The initial value for the non-linear least squares is also important for the proposed positioning method. The result of the initial value convergence analysis shows that initial values that converge to a proper position for all measurement points exist at least with the double array setup.

The positioning theory and simulation methodology described in this work could be a foundation to make a guideline for future applications. For example, the simulation introduced in Section 5 can be used to test the positioning performance of a designed system in advance of installing it, and the initial value analysis shown in Section 6 should be conducted for a different type of antenna setup constellation. In future work, an actual three-dimensional positioning system will be developed and evaluated assuming a practical application.
